# Time trends in occupational exposure to chemicals in Sweden: proportion exposed, distribution across demographic and labor market strata, and exposure levels

**DOI:** 10.5271/sjweh.4040

**Published:** 2022-08-31

**Authors:** Per Gustavsson, Pernilla Wiebert, Håkan Tinnerberg, Theo Bodin, Anette Linnersjö, Ida Hed Myrberg, Maria Albin, Jenny Selander

**Affiliations:** 1 Unit of Occupational Medicine, Institute of Environmental Medicine, Karolinska Institutet, Stockholm, Sweden; 2 Centre for Occupational and Environmental Medicine, Region Stockholm, Stockholm, Sweden; 3 School of Public Health and Community Medicine, Gothenburg University, Gothenburg, Sweden; 4 Division of Biostatistics, Institute of Environmental Medicine, Karolinska Institutet, Stockholm, Sweden

**Keywords:** chlorinated hydrocarbon solvent, construction sector, diesel engine exhaust, JEM, job exposure matrix, lead, migrant worker, respirable crystalline silica, small company, welding fume, wood dust

## Abstract

**Objective:**

This study investigated time trends in occupational exposure to various chemicals in Sweden and the distribution across demographic and labor market sectors.

**Methods:**

Exposure to six chemicals was investigated from 1980 to 2013 by application of a job exposure matrix to national population registers. Respirable crystalline silica (RCS), diesel engine exhaust, welding fumes, wood dust, chlorinated hydrocarbon solvents, and lead were selected to represent different groups of chemicals. Trends in exposure prevalence were investigated by linear regression and compared to the occupationally active population. Confidence intervals for the rate of change over time were obtained by bootstrapping.

**Results:**

The proportion of workers born outside the Nordic countries increased over time in those exposed to RCS, diesel exhaust and wood dust. There was a shift of exposed jobs to small companies (<50 employees), especially for RCS, welding fumes, wood dust, and chlorinated hydrocarbon solvents. For RCS and welding fumes, there was a marked drop in exposure levels from 1980 to 1990 but small changes thereafter. Exposure to lead diminished, both in terms of prevalence and intensity.

**Conclusions:**

Over time, several exposures tended to shift to small companies, the construction sector, and migrant workers, all factors being indicative of less well-controlled working conditions. Occupational exposure to chlorinated organic solvents and lead diminished, while exposure levels to RCS and welding fumes have changed little since 1990. In view of the serious and well-established negative health effects, increased efforts to reduce exposure to RCS and welding fumes are needed.

Working conditions continuously change depending on social, technological, and economic developments. Improvements in legislation, occupational hygiene, and technical improvements in production tend to reduce hazardous exposures, while globalization, segregation of the labor market, and – seen in a global perspective – outsourcing of high exposed jobs to subcontractors or countries outside Europe all may act in the other direction (1).

The few available systematic studies of time trends in occupational chemical exposure have focused more on exposure levels in specific occupations than on prevalence and distribution of exposure in the population. Kauppinen et al (2) used the Finnish job exposure matrix (FINJEM) to estimate the proportion exposed and average exposure levels for the population in Finland from 1950 to 2008. Generally, prevalence of exposure tended to increase up to 1960 and then started to decline. Symanski et al (3) analyzed time trends in exposure levels and found an annual average reduction in exposure levels of 8%. Creely et al (4) reviewed studies of trends in occupational exposure to chemicals and found that levels of vapors and gases changed annually from -24% to +8%. A study of levels of respirable dust and quartz in the European industrial minerals sector 2002–2016 showed an over-all declining trend in exposure levels, but a slight increase during the period 2008–2012, attributed to the global economic crisis (5).

Thus, available studies show declining exposure prevalence and level of chemicals at the workplace. Earlier studies have applied mathematical modelling and estimated the annual decline of exposure levels. However, such modelling may obscure non-logarithmic trends and are not valid beyond the time period on which the data were based. Exposures may both increase and diminish. A study of historical exposure to diesel motor exhaust in Sweden found that occupational exposure levels increased considerably from 1950 to 1970, where after they declined (6). There were similar findings in the study by Kauppinen et al (2).

To our knowledge, no earlier studies have investigated time trends in distribution of exposure across demographic strata as age, gender, and country of birth, or labor market strata as company size, private/public sector, and industrial branches. Such analyses are important for understanding the effects of earlier preventive priorities, for targeting future prevention, and to investigate if exposures tend to accumulate in certain subgroups of the population. Representative data of exposure trends in various strata of the population are key to policies such as the reduction of occupational cancers and the elimination of work-related deaths, outlined in the new European strategy on health and safety at work (7).

The aims of this study were to investigate (i) how exposure to certain specific substances have developed over time in Sweden; (ii) to what extent the exposures have aggregated in demographic or labor market strata in which working conditions may be less strictly controlled; (iii) how exposure prevalence in the general occupationally active population developed over time; and (iv) the average exposure levels in exposed occupations. In order to present sufficient in-depth analyses for each agent, we selected a limited number of chemicals, representing different groups of occupational exposures.

## Methods

This study was based on a linkage of Swedish population registers, holding data on occupational titles, demographic, and labor market factors, with a set of job exposure matrices (JEM). For the years 1980, 1985 and 1990 data on demographic factors and occupation were obtained from the census the respective year. For the period 2001–2013, annual data on occupation, demographic factors, and labor market characteristic were obtained from a longitudinal integrated database for health insurance and labor market studies in Sweden (LISA). Both registers are held by Statistics Sweden. The study was based on all residents in Sweden aged 18–64 years who were occupationally active each year of the study, ie, all persons with a code for occupation and any salary from paid work, disregarding benefits. This group is in the following referred to as the “general population”. The study was designed as a total investigation by a series of total cross-sections of all occupationally active residents in Sweden.

The JEM were originally based on FINJEM (8) and later updates (9). Adaptation to Swedish working conditions was first made for the Nordic Occupational Cancer project (NOCCA) (10). Wiebert et al (11), Videnros et al (12), Graff et al (13) performed additional refinements to reflect Swedish working conditions, and we did the same also for the present study. For an occupation to be included in FINJEM, there was an initial criteria of a presumed exposure prevalence of ≥5%.

For the years 1980, 1985 and 1990, the JEM were coded according to the Swedish occupational coding system NYK (14). For the years 2001–2013, the JEM were coded according to the Swedish occupational coding system SSYK 96, based on ISCO 88 (15).

The following substances were selected for the study, considering both exposure prevalence and health effects of the selected substances. *Respirable crystalline silica (RCS, quartz)* was selected from the group of inorganic particles; *diesel engine exhaust* from combustion-generated particles; *welding fumes* as a complex mixture of gases and particles with wide-spread exposure; *wood dust* was selected from organic particles; from the large group of organic solvents we selected *chlorinated hydrocarbons solvents;* and from metals we selected *lead*. Quantitative exposure to RCS was expressed in mg/m^3^; diesel engine exhaust in mg of NO_2_/m^3^, welding fumes in mg/m^3^, wood dust as inhalable dust in mg/m^3^, chlorinated hydrocarbons solvents in ppm, and lead as µmol/L in blood.

The Ethical Review Board in Stockholm, approved the study (dnr 2018/1007-31/5 and 2019-01704).

### Data analysis

The censuses for the years 1980, 1985 and 1990 had individual data on occupational titles as well as background demographic factors but was not sufficiently detailed data on labor market characteristics. The LISA register held annual individual data on occupational titles as well as demographic and labor market characteristics. Thus, analyses of labor market strata were limited to 2001–2013, while occupational exposures were analyzed for 1980–2013. The variable “company size” was created by counting number of unique individuals per company, the other variables were obtained directly from the register.

The exposure in the population was obtained by linking the JEM to the cohort by occupational titles. The exposure prevalence in the population was obtained by adding the contribution of number of exposed persons (ie, the number of persons in the occupation multiplied by the exposure prevalence) from each exposed occupation and relating it to the total number of persons for each stratum. The average exposure level (in the exposed) was obtained as an average of the contributions of exposure intensity from each exposed occupation, weighed by the number of exposed persons in that occupation. The JEM were specific for occupation and time period but not for gender or other demographic factors.

For the period 1980–1990, the matrices specified exposure with a resolution of ten years. The estimates for 1980 were obtained from the matrices’ estimate for the period 1975–1984, and the estimates for 1990 from the matrix for 1985–1994. For 1985, we used the average between these two time periods. For the period 2001–2013, the matrices had a resolution of three years, and the annual exposure was calculated as a three-year moving average.

The change in percentage points per ten years of the proportion exposed to each chemical was calculated by linear regression, repeated for each exposure and demographic and labor market stratum. A similar model was also fitted to the total occupationally active population. The absolute difference in change in percentage points per ten years between each exposure and the occupationally active population was calculated in order to investigate which exposures that deviated most from the trends in the general population. The statistical significance in difference in time trend for each exposure versus the general population was investigated and confidence intervals (CI) for the slope estimates were obtained by bootstrapping. The time trend for each exposure was assessed as deviating significantly from the average for the general population if beta for the population (taken as a point estimate without variance) fell outside a 95% CI for the exposed group.

Preparation of the data was performed with SAS 9.4 (SAS Institute, Cary, NC, USA) and statistical analysis was performed with R version 4.0.3 (16).

## Results

### Demographic distributions

**Figure 1:**
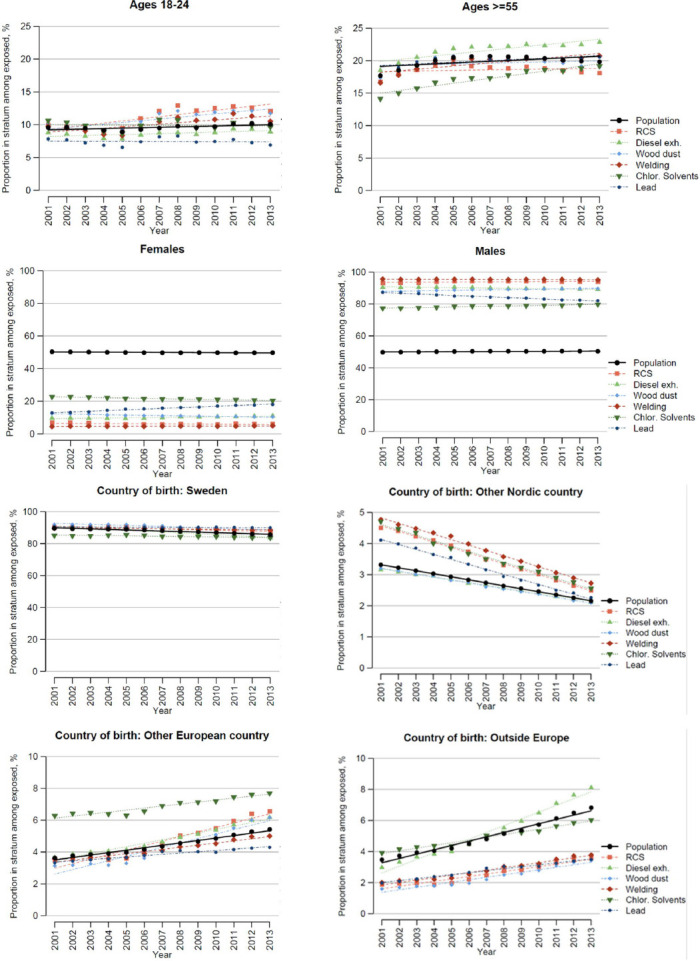
Distribution of exposed persons and the total population across strata of age, sex, and country of birth. Each graph shows how large proportion of those exposed to each chemical that falls into the respective stratum (coloured dots), and the corresponding proportion of the general population (black dots), over time.

**Figure 2:**
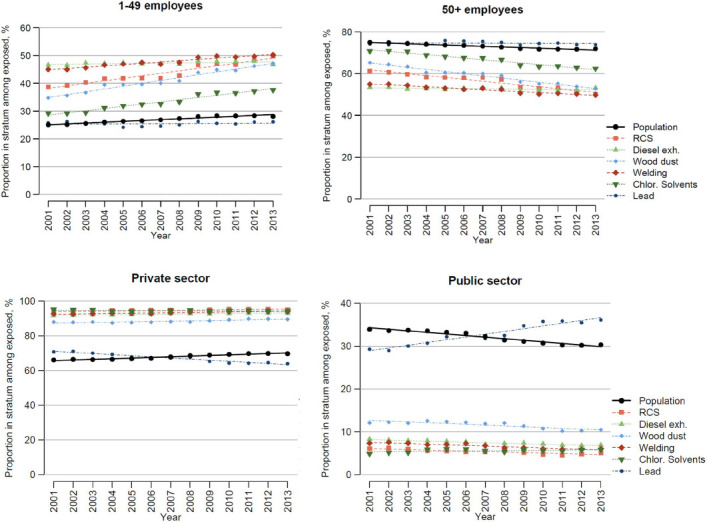
Distribution of exposed persons and the general population across strata of company size and sector. Each graph shows what proportion of those exposed to each chemical that falls into the respective stratum (coloured dotted lines), and the corresponding proportion in the population (black solid line) over time.

The general population that was occupationally active increased from about 3.7 million in 1980 to 4.4 million in 2013 (table 1). The proportion of young workers (18–24 years of age) varied over the years but with no clear trend, both for the general population and in the exposed (figure 1, table 1). The proportion of persons aged >55 years increased slightly in the population (figure 1), and this proportion increased more in those exposed, especially for diesel exhaust and chlorinated solvents (figure 2 and supplementary material, www.sjweh.fi/article/4040, table S1). The difference in trend for these exposures versus the general population was statistically significant (supplementary table S2).

**Table 1:** Characteristics of study population (persons with salary >0 and a code for occupation) over time.

Variables and strata	1980		1985		1990		2001		2005		2009		2013	
						
	N	%	N	%	N	%	N	%	N	%	N	%	N	%
Total study population	3 673 337	100	3 928 931	100	4 140 302	100	3 892 224	100	4 082 073	100	4 218 508	100	4 355 759	100
Age (years)														
18–24	525 081	14.3	604 396	15.4	615 339	14.9	371 467	9.5	363 484	8.9	402 489	9.5	437 870	10.1
25–34	969 016	26.4	947 309	24.1	1 000 773	24.2	925 606	23.8	915 075	22.4	893 503	21.2	949 836	21.8
35–44	897 050	24.4	1 082 537	27.6	1 068 791	25.8	969 688	24.9	1 039 837	25.5	1 076 853	25.5	1 043 789	24
45–54	706 453	19.2	743 500	18.9	917 502	22.2	937 388	24.1	927 421	22.7	978 534	23.2	1 061 285	24.4
≥55	575 737	15.7	551 189	14	537 897	13	688 075	17.7	836 256	20.5	867 129	20.6	862 979	19.8
Sex														
Women	1 681 664	45.8	1 894 373	48.2	2 021 282	48.8	1 956 223	50.3	2 035 060	49.9	2 099 786	49.8	2 166 193	49.7
Men	1 991 673	54.2	2 034 558	51.8	2 119 020	51.2	1 936 001	49.7	2 047 013	50.1	2 118 722	50.2	2 189 566	50.3
Country of birth														
Sweden	3 329 189	90.6	3 567 423	90.8	3 734 833	90.2	3 487 110	89.6	3 629 361	88.9	3 688 028	87.4	3 729 329	85.6
Other Nordic country	204 179	5.6	198 807	5.1	194 835	4.7	129 005	3.3	119 660	2.9	107 684	2.6	93 710	2.2
Other European country	113 579	3.1	121 057	3.1	126 037	3	141 177	3.6	161 871	4	196 451	4.7	235 688	5.4
Other	26 200	0.7	41 515	1.1	84 504	2	134 869	3.5	171 102	4.2	226 245	5.4	296 852	6.8
Company size (employees)														
1–49					990 604	23.9	973 900	25.1	1 073 193	26.3	1 181 055	28	1 220 216	28.1
≥50					3 149 698	76.1	2 911 966	74.9	3 000 362	73.7	3 030 584	72	3 124 837	71.9
Sector														
Private	2 264 969	61.8	2 274 295	58.3	2 507 060	60.6	2 567 601	66.1	2 719 803	66.8	2 901 411	68.9	3 027 559	69.7
Public	1 400 076	38.2	1 628 520	41.7	1 630 917	39.4	1 318 265	33.9	1 353 752	33.2	1 310 228	31.1	1 317 494	30.3
Industry branch														
Not specified					60 901	1.5	51 535	1.3	46 151	1.1	40 346	1	45 415	1
Agriculture, forestry and fishing					84 180	2	41 354	1.1	45 183	1.1	45 451	1.1	40 415	0.9
Manufacturing, mining, and quarrying					879 729	21.2	725 305	18.6	703 993	17.2	629 716	14.9	552 289	12.7
Energy, water and waste supply					42 445	1	34 061	0.9	40 867	1	42 852	1	46 256	1.1
Construction					286 622	6.9	205 453	5.3	221 358	5.4	254 316	6	273 541	6.3
Trade and communication					781 892	18.9	694 904	17.9	749 516	18.4	769 112	18.2	915 573	21
Financial and business activities					404 804	9.8	523 401	13.4	549 376	13.5	658 951	15.6	653 576	15
Education and research					282 242	6.8	358 066	9.2	486 989	11.9	492 217	11.7	492 143	11.3
Health and social work					864 795	20.9	783 782	20.1	713 021	17.5	731 485	17.3	753 991	17.3
Personal and cultural services					233 084	5.6	248 107	6.4	283 345	6.9	309 755	7.3	317 758	7.3
Public administration etc					219 608	5.3	226 256	5.8	242 274	5.9	244 307	5.8	264 802	6.1

The proportion of women in the general population increased from 46% in 1980 to almost 50% in 2013 (table 1). A much lower proportion of women was found for all six exposures. The analysis of absolute difference in change over time in the proportion of women showed deviating results for lead, supplementary table S1, where the proportion of lead-exposed women increased markedly (figure 1). This increase was statistically significantly different from the stable proportion in the population (supplementary table S2). Several of the other exposures also showed significant but smaller deviations from the trend in the population.

A large majority both in the general population and in those exposed were born in Sweden (figure 1). The proportion born in other European countries (outside Nordic countries) increased markedly over time, both in the population and for all six exposures, but this trend was significantly more expressed for RCS, diesel exhaust and wood dust (figure 1, supplementary table S2). The proportion born outside Europe increased in the general population, and this trend was significantly more pronounced for those exposed to diesel exhaust (figure 1, supplementary table S2).

### Labor market strata

The proportion of the general population employed in small companies (<50 employees) increased slightly over the period 2001–2013, figure 2. This trend was more pronounced for those exposed to RCS, welding fumes, wood dust, and chlorinated organic solvents (figure 2). Generally, the proportion employed in small companies was higher than in the general population for all exposures except for lead (figure 2) and the slope of change over time differed significantly from the slope in the population for all six exposures (supplementary table S2).

Employment in the private sector dominated both for the general population and for all six exposures and was considerably more common for all exposures except for lead (figure 2). The proportion of the general population employed in the private sector increased, but the trend over time in transfer from public to private sector was less pronounced for all six exposures than in the population, and employment in private sector actually decreased for lead (figure 2, supplementary table S1). The slope of the change over time differed significantly from that in the population for all six exposures (supplementary table S8).

**Figure 3:**
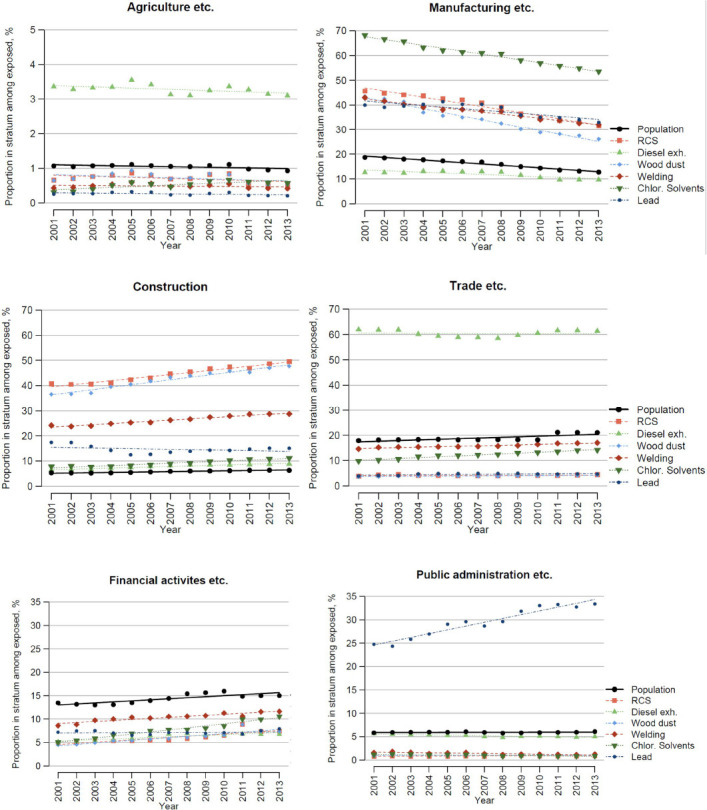
Distribution of exposed persons and the general population across industry branches. Each graph shows how large proportion of those exposed to each chemical that falls into the respective industry branch (coloured dotted lines), and the corresponding proportion in the population (black solid line) over time.

A strong trend was noted in transfer from the manufacturing to construction sectors for those exposed to RCS and wood dust (figure 3, supplementary tables S1 and S4). Exposure to chlorinated organic solvents shifted from manufacturing to the financial activities etc group, which includes activities outsourced to subcontractors. In workers exposed to diesel exhaust, there was an overrepresentation in trade, but a small trend over time (figure 3). For lead, there was a high proportion in public administration and this proportion increased markedly over time (figure 3). Those exposed to diesel exhaust were more often employed in agriculture than were those in the general population as well as those exposed to the other factors (figure 3).

### Prevalence and level of exposures in the population

The prevalence of exposure to RCS, diesel exhaust and welding fumes decreased somewhat over time (supplementary figure S1, tables S1–3). The exposure prevalence was much lower among women than men. For RCS and welding fumes, there was a drop in exposure levels between 1980 and 1990 and smaller reduction since then. Diesel engine exhaust levels showed an almost linear decrease over time (supplementary figure S1). The exposure levels of diesel exhaust and welding fumes were lower for women than men, while surprisingly higher levels of RCS were noted for women than for men.

The proportion exposed to wood dust was fairly stable from 1980–2013 (supplementary figure S1, tables S4–6). There was a large drop in exposure levels between 1990–2001, but the average exposure levels remained essentially unchanged after 2001. The proportion exposed to chlorinated hydrocarbon solvents dropped over the study period, and there was a considerable drop in average exposure levels (supplementary figure S2). The proportion exposed to lead in men dropped markedly from 1980 to 2013. Lead levels in blood dropped almost linearly in men, and with further reduced lead levels in women since 2001.

## Discussion

### Demographic and labor market distribution

The analysis of exposure across demographic strata showed that workers exposed to diesel exhaust and chlorinated hydrocarbon solvents tended to increase in workers 55 years of age or more (figure 1). Another important finding, discussed further below, was that the proportion of women increased among those exposed to lead (figure 1 and supplementary table S8).

The proportion of women in the general occupationally active population was close to 50%, but all six exposures was much more common among men than women (figure 1). However, the JEM were not gender-specific, and the analysis of exposure levels among men and women rather reflected how men and women were distributed over occupations with low or high exposures. This analysis surprisingly indicated higher exposure levels among women versus men for RCS. This may be due to an underrepresentation of women in the large construction sector, in which there is a large number of low- or medium-exposed persons, mainly men, contributing to a lower average exposure level among exposed men than among women, although much fewer women than men are exposed.

Most of the exposed workers were born in Sweden. However, there was a strong trend of an increasing proportion of workers born outside the Nordic countries over the study period (figure 1). The proportion exposed to RCS, diesel exhaust and wood dust increased significantly more in those born in Europe outside the Nordic countries than in the general population (supplementary table S8). Being born outside Europe was associated with an increasing proportion exposed to diesel exhaust. A recent systematic review showed that being foreign-born is associated with poorer employment conditions, leading to a higher risk of uncontrolled exposure and negative health effects (17).

There was a general trend in the workforce of transfer from large to small-sized companies. In Sweden, a safety committee is not mandatory for companies with <50 employees, and small companies have less resources for occupational safety and health (18). For RCS, wood dust and chlorinated solvents there was a marked and statistically significant transfer to small-sized companies (figure 2 and supplementary table S1–2). The analysis of private/public sector showed a strong accumulation of lead-exposed in the public sector (figure 2). There was a similar finding in the analysis of industry branch, which showed an accumulation of exposure to lead in Public administration (figure 3 and supplementary table S8). This combination of findings is likely related to an increasing number of female police officers, and that lead exposure during shooting practice has been recognized as a lead-exposed situation under mandatory monitoring (19). This exposure may present a health hazard especially regarding neurodevelopmental disorders for the fetus of pregnant women (20). Even if relatively few women are occupationally exposed to lead, it is important to keep women in reproductive ages under close surveillance for potential exposure to lead.

The trend of exposure to RCS and wood dust to move from manufacturing to construction is partly related to a general growth of the latter sector, in which exposure to RSC and wood dust are highly prevalent. This in not only a shift of relative proportions but also a shift in actual numbers of exposed workers (supplementary tables S1 and S4). Employment in the construction sector is associated with extra health risk not only from accidents and physical hazards but also from chemical exposures (21). The construction sector is characterized by many small-sized subcontractor firms and an increasing proportion of immigrant workers (22), both associated with poorer working conditions (17, 18). The construction industry also has many temporary job sites, shown to be associated with a larger variability in exposure than permanent jobs (23).

### Exposure prevalence and level

The exposure prevalence generally diminished, but this was clearly marked only for chlorinated hydrocarbon solvents and lead, while the exposure prevalence was almost unchanged since 1980 for wood dust and decreased only slightly for RCS, diesel exhaust, and welding fumes. Average exposure levels in the exposed were reduced for all six exposure factors. However, most of the reduction for RCS and welding fumes took place before 1990, and exposures were reduced only marginally thereafter. Possibly, this reflects a period of intensive focus on reducing chemical workplace hazards in Sweden in the 1970s and 80s (24).

As estimated from this study, the progress in reducing exposure to RCS since the shift of the millennium up to 2013 was, at best, marginal. There were similar findings in a study of exposure to RCS in the European industrial minerals sector, indicated rising exposure levels in 2008–2012 (5). Similarly, in Australian mining exposure levels were reduced from 1985 up to about 2005 and then increased slightly (25). The absolute average exposure level for RCS predicted by the JEM in 2013 was just <50 µg/m^3^, fig S1. This is in close correspondence with a current study from Denmark, showing an arithmetic average of 46 µg of quartz/m^3^, based on 189 full-shift samples in quartz-exposed occupations in Denmark in 2018 (26).

Kauppinen et al (2) projected exposure prevalence and exposure levels in Finland up to 2020. They found only marginally reduced exposure prevalence for RCS and diesel engine exhaust from 1990–2020, which is in accordance with the findings from the present study. A JEM using elemental carbon as indicator for diesel exhaust in Sweden (not used for exposure estimations in the present study) showed dramatic reductions in exposure from 1970 up to about 1990 and smaller reduction thereafter (6).

Our analysis showed a very small reduction of welding fume levels after 1990, while the analysis by Kauppinen et al (2) showed a slightly more pronounced reduction for Finland. Welding fumes have recently been classified as carcinogenic to humans by the IARC (27) and is also associated with cardiovascular as well as respiratory disease. Most countries are lacking a specific exposure limit value for welding fumes, which is urgently needed (28).

The general picture of minor improvements regarding exposure levels for RCS, diesel exhaust and welding fumes is of serious concern, since these three exposures have been pointed out as large contributors to the current burden of occupational disease. They now account for about half of the population attributable fraction for occupational lung cancer (29), which earlier was largely dominated by asbestos. In addition, there is evidence that these three exposures also cause ischemic heart disease (30) as well as COPD (31). Thus, RCS, diesel exhaust and welding fumes are large contributors to occupationally induced chronic disease and mortality, and further exposure reduction is a priority in view of national and European strategies to eliminate deaths caused by work (7).

For wood dust, we observed a very small reduction in exposure prevalence, but a large reduction in average exposure levels between 1990–2001, and almost stable levels thereafter. An analysis of trends in exposure to wood dust in the UK found an average annual reduction in exposure of about 8% from 1985–2005 (32).

Clear reductions of exposure to chlorinated organic solvents were observed, both in terms of exposure prevalence and average exposure level. This is in accordance with the findings in Finland (2). The reduced exposures may be related both to improved exposure control and the replacement of organic solvents with water-based products.

The prevalence of exposure to lead decreased markedly from 1980 to 2001, and the average exposure level also declined. A decreasing trend of blood lead levels was reported in lead-exposed industrial workers in the US, showing annual declines of 2–11%, based on data mainly derived from measurements in 1950–2000 (33). It is positive that the exposure prevalence is diminishing, but the negative health effects for those still exposed is of concern.

### Strengths and weaknesses

This study has an advantage in its large size and is an investigation of the entire population of occupationally active residents in Sweden. Thus, there is no fluctuations by chance. We investigated statistical significance of trends in order to investigate to what extent trends in occupational exposures could be explained by similar trends in the general population.

JEM represent an efficient way of classifying the exposure for large cohorts, where individualized exposure assessment is not possible. However, the application of matrices is associated with misclassification of exposure (34). This misclassification may be of a classical error type, but also due to Berkson errors, not causing attenuation of risks but rather a loss in precision (35). The absolute levels of exposure to RCS corresponded closely with those found in an investigation from Denmark, supporting the validity of the application of the matrices to population data.

One limitation of the present study is that it was not possible to investigate time trends in exposures after 2013. The reason for this is that the occupational codes in the national database shifted from SSYK 96 (based on ISCO 88) to SSYK 12, which is based on ISCO 2008, and that the JEMs were not available in the latter coding system. There are no valid crosswalks between these two coding schemes, and an extension of analyses of trends must wait until new matrices are developed based on ISCO 2008.

Another limitation is that the matrices were not specific for gender (or any other demographic characteristic). Thus, any interaction between demographic variables and exposure trend could not be investigated. Finally, in order to present sufficient in-depth analyses for each agent, we studied a limited number of chemicals. Thus, the trends we have observed cannot be assumed to apply to chemicals in general, although we selected agents to represent major groups of different chemicals.

### Concluding remarks

This study showed that exposure to most of the six studied substances is more common in small companies with smaller resources for preventive work, and that this proportion is growing, especially for RCS, wood dust and chlorinated solvents. Being foreign-born was associated with increasing proportions of exposure to RCS, diesel exhaust and wood dust. Being foreign-born as well as employment in small companies was associated with poorer working conditions. Exposure to RCS and wood dust shifted from manufacturing to the construction sector, a trade with many temporary job sites and less well controlled working conditions. Exposure to lead during pregnancy is of concern, and – even if relatively few women are exposed – the negative health effects for the fetuses are serious.

This study confirmed some general reductions both in exposure prevalence and levels, but that much of the reductions took place in the 1980s, with less progress since 1990. Prevention of harmful occupational chemical exposures seem to have been more successful for chlorinated organic solvents and lead, while exposure to RCS, welding fumes and possibly also diesel exhaust have changed less. As mentioned above, exposure to RCS and diesel exhaust has transferred to sectors with less well controlled working conditions. In view of the serious and well-established negative health effects and growing evidence of multiple diseases associated with these exposures, more emphasis is needed in reducing exposure to these agents.

## Supplementary material

Supplementary material
