# Supplementary material for: Time trends in occupational exposure to chemicals in Sweden: proportion exposed, distribution across demographic and labor market strata, and exposure levels
Source: Scand J Work Environ Health. 2022 Aug 31;48(6):479–89. doi: 10.5271/sjweh.4040 (PMC9888437; doi:10.5271/sjweh.4040)
Supplement: Supplementary material [file SJWEH-48-479-S001.pdf]

Time trends in occupational exposure to chemicals in Sweden: proportion exposed, distribution across demographic and labor market strata, and exposure levels[1]

by Per Gustavsson, MD,[2] Pernilla Wiebert, PhD, Håkan Tinnerberg, PhD, Theo Bodin, MD, Anette Linnersjö, Lic Med Sci, Ida Hed Myrberg, MSc, Maria Albin, MD, Jenny Selander, PhD

1. Supplementary material
2. Correspondence to: Per Gustavsson, Unit of Occupational Medicine, Karolinska Institutet, Solnavägen 4, 113 65 Stockholm, Sweden. [E-mail: per.gustavsson@ki.se]

Table S1. Distribution of persons exposed to respirable crystalline silica (RCS) across demographic and labour market strata and over time.

	1980		1985		1990		2001		2005		2009		2013	
**Stratum**	**n**	**%**	**n**	**%**	**n**	**%**	**n**	**%**	**n**	**%**	**n**	**%**	**n**	**%**
**Exposed in study population**	146 150	100	130 755	100	142 082	100	102 960	100	106 456	100	113 250	100	116 020	100
**Age**														
18-24	24 803	17	24 124	18.4	27 110	19.1	10 233	9.9	10 078	9.5	13 754	12.1	14 034	12.1
25-34	37 887	25.9	29 984	22.9	37 692	26.5	25 288	24.6	23 516	22.1	23 410	20.7	26 780	23.1
35-44	32 436	22.2	33 860	25.9	33 045	23.3	27 005	26.2	28 863	27.1	28 690	25.3	25 655	22.1
45-54	26 359	18	24 801	19	28 270	19.9	23 255	22.6	23 227	21.8	25 793	22.8	28 558	24.6
>=55	24 673	16.9	17 991	13.8	15 966	11.2	17 182	16.7	20 775	19.5	21 605	19.1	20 994	18.1
**Sex**														
Females	6 060	4.1	5 125	3.9	5 375	3.8	6 993	6.8	6 069	5.7	6 434	5.7	6 899	5.9
Males	140 094	95.9	125 630	96.1	136 709	96.2	95 965	93.2	100 387	94.3	106 817	94.3	109 120	94.1
**Country of birth**														
Sweden	132 583	90.7	119 749	91.6	130 292	91.7	92 809	90.1	96 339	90.5	100 624	88.9	101 372	87.4
Other Nordic country	10 435	7.1	8 285	6.3	8 190	5.8	4 640	4.5	4 192	3.9	3 593	3.2	2 893	2.5
Other European country	2 689	1.8	2 296	1.8	2 430	1.7	3 593	3.5	3 831	3.6	5 857	5.2	7 592	6.5
Outside Europe	428	0.3	426	0.3	1 174	0.8	1 916	1.9	2 093	2	3 173	2.8	4 152	3.6
**Company size**														
1-49 employees					48 040	33.8	39 814	38.7	44 483	41.8	52 635	46.5	57 553	49.7
50+ employees					94 043	66.2	63 035	61.3	61 822	58.2	60 497	53.5	58 188	50.3
**Sector**														
Private	128 550	88.2	113 476	87.6	129 930	91.5	96 626	93.9	100 523	94.6	107 318	94.9	109 864	94.9
Public	17 201	11.8	16 021	12.4	12 078	8.5	6 225	6.1	5 783	5.4	5 812	5.1	5 874	5.1

**Industry branch**										
Not specified	2 008	1.4	602	0.6	740	0.7	1 275	1.1	2 176	1.9
Agriculture etc.	1 255	0.9	670	0.7	915	0.9	925	0.8	598	0.5
Manufacturing etc.	31 712	22.3	46 980	45.6	45 308	42.6	41 278	36.4	36 407	31.4
Energy supply	4 449	3.1	551	0.5	619	0.6	832	0.7	818	0.7
Construction	89 115	62.7	41 898	40.7	45 002	42.3	52 780	46.6	57 443	49.5
Trade etc.	4 303	3	3 978	3.9	4 393	4.1	4 576	4	5 193	4.5
Financial activities etc.	5 348	3.8	5 069	4.9	5 642	5.3	6 912	6.1	8 552	7.4
Education etc.	415	0.3	454	0.4	575	0.5	686	0.6	830	0.7
Health etc.	1 392	1	502	0.5	560	0.5	746	0.7	885	0.8
Personal services etc.	1 250	0.9	1 463	1.4	1 909	1.8	2 159	1.9	2 077	1.8
Public administration etc.	850	0.6	797	0.8	799	0.8	1 083	1	1 048	0.9

Table S2. Distribution of persons exposed to diesel exhaust across demographic and labour market strata and over time

	1980		1985		1990		2001		2005		2009		2013	
**Stratum**	**n**	**%**	**n**	**%**	**n**	**%**	**n**	**%**	**n**	**%**	**n**	**%**	**n**	**%**
**Exposed in study population**	147 169	100	137 599	100	146 255	100	105 108	100	114 861	100	119 446	100	118 309	100
**Age**														
18-24	20 890	14.2	17 665	12.8	19 485	13.3	9 222	8.8	8 978	7.8	10 142	8.5	10 517	8.9
25-34	42 703	29	35 941	26.1	38 054	26	22 654	21.6	23 397	20.4	24 293	20.3	25 439	21.5
35-44	37 787	25.7	40 200	29.2	37 737	25.8	27 693	26.3	29 284	25.5	27 724	23.2	24 592	20.8
45-54	26 643	18.1	26 794	19.5	33 630	23	26 112	24.8	28 107	24.5	30 420	25.5	30 743	26
>=55	19 157	13	17 003	12.4	17 356	11.9	19 431	18.5	25 101	21.9	26 863	22.5	27 017	22.8
**Sex**														
Females	10 853	7.4	11 306	8.2	12 829	8.8	10 064	9.6	10 818	9.4	12 370	10.4	12 950	10.9
Males	136 323	92.6	126 295	91.8	133 427	91.2	95 046	90.4	104 043	90.6	107 077	89.6	105 355	89.1
**Country of birth**														
Sweden	134 569	91.4	126 159	91.7	133 332	91.2	94 837	90.2	102 258	89	103 239	86.4	98 911	83.6
Other Nordic country	7 854	5.3	6 815	5	6 949	4.8	3 320	3.2	3 275	2.9	2 985	2.5	2 494	2.1
Other European country	3 835	2.6	3 623	2.6	3 971	2.7	3 831	3.6	4 741	4.1	6 115	5.1	7 307	6.2
Outside Europe	910	0.6	1 002	0.7	2 003	1.4	3 123	3	4 584	4	7 104	5.9	9 590	8.1
**Company size**														
1-49 employees					56 839	38.9	48 896	46.6	54 402	47.5	57 427	48.1	55 183	46.7
50+ employees					89 419	61.1	56 069	53.4	60 233	52.5	61 879	51.9	62 875	53.3
**Sector**														
Private	125 897	85.7	114 858	84.2	126 964	86.9	96 314	91.8	105 780	92.3	110 508	92.6	109 902	93.1
Public	21 002	14.3	21 548	15.8	19 181	13.1	8 650	8.2	8 853	7.7	8 799	7.4	8 154	6.9
**Industry branch**														
Not specified					1 867	1.3	634	0.6	735	0.6	615	0.5	666	0.6
Agriculture etc.					1 464	1	3 531	3.4	4 077	3.5	3 877	3.2	3 662	3.1
Manufacturing etc.					35 722	24.4	13 219	12.6	15 085	13.1	13 718	11.5	11 363	9.6
Energy supply					2 746	1.9	1 368	1.3	1 821	1.6	1 900	1.6	1 763	1.5
Construction					19 901	13.6	6 799	6.5	8 577	7.5	10 057	8.4	10 409	8.8
Trade etc.					71 206	48.7	65 113	61.9	68 186	59.4	71 312	59.7	72 620	61.4
Financial activities etc.					4 478	3.1	5 354	5.1	6 698	5.8	7 822	6.5	7 976	6.7
Education etc.					566	0.4	733	0.7	792	0.7	874	0.7	644	0.5
Health etc.					1 654	1.1	848	0.8	920	0.8	1 192	1	1 237	1
Personal services etc.					1 339	0.9	1 606	1.5	1 909	1.7	2 082	1.7	2 072	1.8
Public administration etc.					5 317	3.6	5 904	5.6	6 066	5.3	5 996	5	5 893	5

Table S3. Distribution of persons exposed to welding fumes across demographic and labour market strata and over time.

	1980		1985		1990		2001		2005		2009		2013	
**Stratum**	n	%	n	%	n	%	n	%	n	%	n	%	n	%
**Exposed in study population**	111 306	100	102 336	100	99 962	100	71 644	100	70 356	100	71 219	100	70 306	100
**Age**														
18-24	22 801	20.5	21 525	21	20 924	20.9	6 970	9.7	5 860	8.3	7 573	10.6	7 384	10.5
25-34	32 432	29.1	27 098	26.5	26 764	26.8	17 629	24.6	14 902	21.2	14 237	20	15 061	21.4
35-44	25 968	23.3	26 055	25.5	23 285	23.3	18 801	26.2	19 048	27.1	17 872	25.1	15 386	21.9
45-54	17 064	15.3	17 126	16.7	19 320	19.3	16 358	22.8	16 303	23.2	17 059	24	17 913	25.5
>=55	13 047	11.7	10 536	10.3	9 667	9.7	11 884	16.6	14 241	20.2	14 476	20.3	14 563	20.7
**Sex**														
Females	6 992	6.3	6 788	6.6	7 797	7.8	3 124	4.4	3 147	4.5	3 201	4.5	3 417	4.9
Males	104 312	93.7	95 549	93.4	92 166	92.2	68 518	95.6	67 209	95.5	68 016	95.5	66 891	95.1
**Country of birth**														
Sweden	93 930	84.4	88 534	86.5	86 110	86.1	64 242	89.7	63 191	89.8	63 444	89.1	62 224	88.5
Other Nordic country	11 671	10.5	8 969	8.8	8 004	8	3 416	4.8	2 983	4.2	2 442	3.4	1 915	2.7
Other European country	4 921	4.4	3 988	3.9	3 680	3.7	2 558	3.6	2 567	3.6	3 134	4.4	3 513	5
Outside Europe	773	0.7	850	0.8	2 167	2.2	1 428	2	1 612	2.3	2 195	3.1	2 651	3.8
**Company size**														
1-49 employees					35 007	35	32 234	45	33 013	47	35 138	49.4	35 259	50.3
50+ employees					64 953	65	39 343	55	37 252	53	36 012	50.6	34 894	49.7
**Sector**														
Private	104 885	94.4	94 800	93.8	94 606	94.7	66 342	92.7	65 331	93	66 689	93.7	65 926	94
Public	6 255	5.6	6 230	6.2	5 318	5.3	5 236	7.3	4 934	7	4 460	6.3	4 227	6
**Industry branch**														
Not specified					1 231	1.2	447	0.6	465	0.7	472	0.7	659	0.9
Agriculture etc.					546	0.5	307	0.4	414	0.6	362	0.5	291	0.4
Manufacturing etc.					59 475	59.5	30 813	43	26 815	38.1	25 376	35.6	22 585	32.1
Energy supply					654	0.7	436	0.6	535	0.8	471	0.7	581	0.8
Construction					17 224	17.2	17 297	24.1	17 775	25.3	19 523	27.4	20 183	28.7
Trade etc.					15 409	15.4	10 427	14.6	10 859	15.4	11 313	15.9	11 950	17
Financial activities etc.					3 041	3	6 148	8.6	7 296	10.4	7 635	10.7	8 162	11.6
Education etc.					225	0.2	1 539	2.1	1 501	2.1	1 347	1.9	1 240	1.8
Health etc.					692	0.7	621	0.9	562	0.8	1 055	1.5	1 099	1.6
Personal services etc.					472	0.5	2 468	3.4	3 070	4.4	2 775	3.9	2 690	3.8
Public administration etc.					997	1	1 138	1.6	1 056	1.5	885	1.2	871	1.2

Table S4. Distribution of persons exposed to wood dust across demographic and labour market strata and over time.

	1980		1985		1990		2001		2005		2009		2013	
**Stratum**	**n**	**%**	**n**	**%**	**n**	**%**	**n**	**%**	**n**	**%**	**n**	**%**	**n**	**%**
**Exposed in study population**	107 211	100	93 134	100	98 282	100	102 710	100	95 126	100	98 527	100	100 661	100
**Age**														
18-24	20 376	19	18 016	19.3	19 292	19.6	10 337	10.1	8 737	9.2	11 383	11.6	11 768	11.7
25-34	28 622	26.7	22 327	24	25 286	25.7	24 812	24.2	21 211	22.3	20 334	20.6	22 767	22.6
35-44	21 423	20	23 295	25	23 733	24.1	26 257	25.6	24 584	25.8	24 464	24.8	22 351	22.2
45-54	17 824	16.6	15 857	17	18 283	18.6	23 491	22.9	20 911	22	22 499	22.8	24 640	24.5
>=55	18 967	17.7	13 640	14.6	11 688	11.9	17 811	17.3	19 685	20.7	19 841	20.1	19 133	19
**Sex**														
Females	9 503	8.9	8 835	9.5	9 572	9.7	12 750	12.4	10 701	11.2	10 606	10.8	10 448	10.4
Males	97 710	91.1	84 299	90.5	88 711	90.3	89 957	87.6	84 423	88.8	87 923	89.2	90 214	89.6
**Country of birth**														
Sweden	99 915	93.2	87 320	93.8	91 470	93.1	94 589	92.1	87 564	92.1	88 942	90.3	88 914	88.3
Other Nordic country	5 238	4.9	3 968	4.3	4 178	4.3	3 287	3.2	2 678	2.8	2 412	2.4	2 104	2.1
Other European country	1 723	1.6	1 469	1.6	1 623	1.7	3 205	3.1	3 125	3.3	4 656	4.7	6 200	6.2
Outside Europe	333	0.3	378	0.4	1 008	1	1 624	1.6	1 755	1.8	2 517	2.6	3 439	3.4
**Company size**														
1-49 employees					37 934	38.6	35 664	34.8	37 552	39.5	43 213	43.9	47 439	47.2
50+ employees					60 347	61.4	66 939	65.2	57 451	60.5	55 222	56.1	53 057	52.8
**Sector**														
Private	98 871	92.4	82 702	90.1	90 813	92.5	90 226	87.9	83 274	87.7	87 283	88.7	89 986	89.5
Public	8 148	7.6	9 095	9.9	7 413	7.5	12 379	12.1	11 728	12.3	11 153	11.3	10 509	10.5
**Industry branch**														
Not specified					1 775	1.8	733	0.7	791	0.8	1 120	1.1	1 922	1.9
Agriculture etc.					1 040	1.1	663	0.6	892	0.9	827	0.8	534	0.5
Manufacturing etc.					40 894	41.6	44 722	43.5	33 920	35.7	29 681	30.1	26 229	26.1
Energy supply					173	0.2	504	0.5	556	0.6	634	0.6	618	0.6
Construction					41 220	41.9	37 493	36.5	38 517	40.5	44 259	44.9	47 990	47.7
Trade etc.					3 790	3.9	3 610	3.5	4 184	4.4	3 905	4	4 356	4.3
Financial activities etc.					3 316	3.4	4 572	4.5	5 105	5.4	6 180	6.3	7 412	7.4
Education etc.					3 089	3.1	7 366	7.2	7 676	8.1	7 988	8.1	7 501	7.5
Health etc.					1 378	1.4	570	0.6	552	0.6	837	0.8	955	0.9
Personal services etc.					938	1	1 628	1.6	2 100	2.2	2 156	2.2	2 143	2.1
Public administration etc.					670	0.7	846	0.8	834	0.9	941	1	1 004	1

Table S5. Distribution of persons exposed to chlorinated organic solvents across demographic and labour market strata and over time.

	1980		1985		1990		2001		2005		2009		2013	
**Stratum**	**n**	**%**	**n**	**%**	**n**	**%**	**n**	**%**	**n**	**%**	**n**	**%**	**n**	**%**
**Exposed in study population**	24 784	100	22 843	100	21 909	100	10 444	100	9 664	100	9 090	100	8 481	100
**Age**														
18-24	4 876	19.7	4 745	20.8	4 243	19.4	1 107	10.6	838	8.7	860	9.5	816	9.6
25-34	6 957	28.1	6 019	26.3	5 822	26.6	2 887	27.6	2 346	24.3	1 952	21.5	1 833	21.6
35-44	5 307	21.4	5 654	24.7	5 380	24.6	2 767	26.5	2 708	28	2 461	27.1	2 037	24
45-54	4 048	16.3	3 733	16.3	4 159	19	2 209	21.1	2 115	21.9	2 144	23.6	2 170	25.6
>=55	3 597	14.5	2 695	11.8	2 307	10.5	1 475	14.1	1 662	17.2	1 675	18.4	1 625	19.2
**Sex**														
Females	6 303	25.4	5 950	26	5 874	26.8	2 372	22.7	2 086	21.6	1 921	21.1	1 724	20.3
Males	18 482	74.6	16 894	74	16 035	73.2	8 073	77.3	7 577	78.4	7 167	78.9	6 756	79.7
**Country of birth**														
Sweden	20 843	84.1	19 622	85.9	18 706	85.4	8 890	85.1	8 267	85.5	7 672	84.4	7 099	83.7
Other Nordic country	2 409	9.7	1 811	7.9	1 507	6.9	492	4.7	372	3.8	293	3.2	217	2.6
Other European country	1 300	5.2	1 099	4.8	1 013	4.6	655	6.3	608	6.3	648	7.1	652	7.7
Outside Europe	229	0.9	311	1.4	680	3.1	408	3.9	420	4.3	474	5.2	511	6
**Company size**														
1-49 employees					5 793	26.4	3 045	29.2	3 074	31.8	3 276	36.1	3 180	37.6
50+ employees					16 117	73.6	7 388	70.8	6 578	68.2	5 804	63.9	5 274	62.4
**Sector**														
Private	21 602	87.2	19 411	85.8	18 966	86.6	9 928	95.1	9 072	94	8 557	94.2	7 965	94.2
Public	3 161	12.8	3 221	14.2	2 935	13.4	508	4.9	577	6	523	5.8	489	5.8
**Industry branch**														
Not specified					277	1.3	46	0.4	50	0.5	45	0.5	48	0.6
Agriculture etc.					117	0.5	32	0.3	57	0.6	57	0.6	48	0.6
Manufacturing etc.					12 686	57.9	7 117	68.1	5 992	62	5 277	58	4 538	53.5
Energy supply					95	0.4	90	0.9	101	1	95	1	91	1.1
Construction					2 076	9.5	812	7.8	785	8.1	888	9.8	940	11.1
Trade etc.					2 413	11	1 021	9.8	1 154	11.9	1 180	13	1 199	14.1
Financial activities etc.					682	3.1	527	5	669	6.9	728	8	895	10.6
Education etc.					737	3.4	149	1.4	196	2	168	1.8	86	1
Health etc.					1 455	6.6	235	2.3	236	2.4	254	2.8	246	2.9
Personal services etc.					1 119	5.1	299	2.9	315	3.3	323	3.6	314	3.7
Public administration etc.					252	1.2	116	1.1	106	1.1	79	0.9	72	0.8

Table S6. Distribution of persons exposed to lead across demographic and labour market strata and over time.

	1980		1985		1990		2001		2005		2009		2013	
**Stratum**	**n**	**%**	**n**	**%**	**n**	**%**	**n**	**%**	**n**	**%**	**n**	**%**	**n**	**%**
**Exposed in study population**	108 215	100	82 487	100	52 594	100	13 098	100	10 869	100	10 451	100	10 303	100
**Age**														
18-24	20 238	18.7	16 266	19.7	9 641	18.3	1 024	7.8	708	6.5	766	7.3	708	6.9
25-34	31 957	29.5	21 583	26.2	13 486	25.6	3 064	23.4	2 333	21.5	2 404	23	2 464	23.9
35-44	25 850	23.9	22 103	26.8	13 094	24.9	3 462	26.4	2 989	27.5	2 740	26.2	2 524	24.5
45-54	17 179	15.9	13 760	16.7	10 997	20.9	3 218	24.6	2 597	23.9	2 403	23	2 488	24.2
>=55	12 997	12	8 773	10.6	5 374	10.2	2 330	17.8	2 242	20.6	2 140	20.5	2 118	20.6
**Sex**														
Females	16 299	15.1	14 274	17.3	9 070	17.2	1 678	12.8	1 639	15.1	1 706	16.3	1 849	17.9
Males	91 918	84.9	68 216	82.7	43 522	82.8	11 417	87.2	9 228	84.9	8 744	83.7	8 452	82.1
**Country of birth**														
Sweden	94 435	87.3	72 732	88.2	46 005	87.5	11 867	90.6	9 822	90.3	9 423	90.2	9 271	90
Other Nordic country	8 829	8.2	5 885	7.1	3 554	6.8	538	4.1	386	3.6	295	2.8	233	2.3
Other European country	4 204	3.9	3 015	3.7	1 865	3.5	434	3.3	397	3.7	420	4	442	4.3
Outside Europe	743	0.7	854	1	1 169	2.2	258	2	268	2.5	314	3	359	3.5
**Company size**														
1-49 employees					12 891	24.5	3 372	25.8	2 623	24.2	2 736	26.2	2 683	26.1
50+ employees					39 702	75.5	9 715	74.2	8 235	75.8	7 711	73.8	7 598	73.9
**Sector**														
Private	84 755	78.4	63 629	77.7	41 454	78.8	9 258	70.7	7 363	67.8	6 815	65.3	6 567	63.9
Public	23 346	21.6	18 210	22.3	11 130	21.2	3 831	29.3	3 496	32.2	3 629	34.7	3 711	36.1
**Industry branch**														
Not specified					448	0.9	73	0.6	55	0.5	46	0.4	68	0.7
Agriculture etc.					148	0.3	33	0.3	35	0.3	28	0.3	21	0.2
Manufacturing etc.					28 080	53.4	5 233	40	4 491	41.3	3 757	36	3 403	33
Energy supply					442	0.8	91	0.7	82	0.8	77	0.7	78	0.8
Construction					6 974	13.3	2 275	17.4	1 350	12.4	1 494	14.3	1 548	15
Trade etc.					12 396	23.6	509	3.9	524	4.8	515	4.9	479	4.7
Financial activities etc.					1 351	2.6	940	7.2	704	6.5	726	6.9	815	7.9
Education etc.					127	0.2	230	1.8	137	1.3	121	1.2	104	1
Health etc.					325	0.6	94	0.7	57	0.5	98	0.9	97	0.9
Personal services etc.					233	0.4	378	2.9	274	2.5	260	2.5	247	2.4
Public administration etc.					2 083	4	3 238	24.7	3 157	29.1	3 325	31.8	3 440	33.4

Table S7. Change in percent points per 10 years in each stratum and the absolute difference of change in exposed vs. population over the period 2001-2013. Light blue: small difference; dark blue: large difference.

	Population	RCS		Diesel exhaust		Wood dust		Welding fumes		Chlor. solvents		Lead	
Variable	Change per 10 y	Change per 10 y	diff vs pop	Change per 10 y	diff vs pop	Change per 10 y	diff vs pop	Change per 10 y	diff vs pop	Change per 10 y	diff vs pop	Change per 10 y	diff vs pop
Ages 18-24	0.66	3.21	2.55	0.73	0.07	2.6	1.94	2.07	1.41	-0.11	0.77	-0.08	0.74
Ages 25-34	-1.93	-1.59	0.34	0.24	2.17	-1.73	0.2	-2.59	0.66	-5.4	3.47	2.02	3.95
Ages 35-44	-0.65	-4.04	3.39	-5.18	4.53	-3.11	2.46	-4.04	3.39	-2.28	1.63	-2.2	1.55
Ages 45-54	0.59	2.02	1.43	1.35	0.76	1.64	1.05	2.11	1.52	4.01	3.42	-1.02	1.61
Ages >=55	1.32	0.41	0.91	2.86	1.54	0.6	0.72	2.47	1.15	3.78	2.46	1.27	0.05
Females	-0.47	-0.66	0.19	1.28	1.75	-1.67	1.2	0.25	0.72	-1.94	1.47	4.44	4.91
Males	0.47	0.66	0.19	-1.28	1.75	1.67	1.2	-0.25	0.72	1.94	1.47	-4.44	4.91
Country of birth: Sweden	-3.36	-2.69	0.67	-5.64	2.28	-3.48	0.12	-1.19	2.17	-1.27	2.09	-0.48	2.88
Country of birth: Other Nordic country	-0.97	-1.74	0.77	-0.88	0.09	-0.93	0.04	-1.75	0.78	-1.74	0.77	-1.61	0.64
Country of birth: Other European c.	1.53	2.85	1.32	2.16	0.63	2.8	1.27	1.34	0.19	1.26	0.27	0.81	0.72
Country of birth: Outside Europe	2.79	1.58	1.21	4.35	1.56	1.61	1.18	1.6	1.19	1.75	1.04	1.27	1.52
1-49 employees	3.07	9.01	5.94	0.76	2.31	10.22	7.15	4.65	1.58	7.95	4.88	0.27	2.8
50+ employees	-3.07	-9.01	5.94	-0.76	2.31	-10.22	7.15	-4.65	1.58	-7.95	4.88	-0.27	2.8
Private sector	3.68	1.19	2.49	1.19	2.49	1.89	1.79	1.49	2.19	-0.46	4.14	-6.43	10.11
Public sector	-3.68	-1.19	2.49	-1.19	2.49	-1.89	1.79	-1.49	2.19	0.46	4.14	6.43	10.11
Industry branch not specified	-0.28	1.08	1.36	-0.1	0.18	0.98	1.26	0.17	0.45	0.15	0.43	0.05	0.33
Agriculture etc.	-0.09	-0.15	0.06	-0.18	0.09	-0.15	0.06	-0.04	0.05	0.23	0.32	-0.05	0.04
Manufacturing etc.	-5.26	-12.49	7.23	-2.99	2.27	-14.95	9.69	-8.63	3.37	-11.74	6.48	-6.21	0.95
Energy supply	0.13	0.15	0.02	0.13	0	0.07	0.06	0.13	0	0.04	0.09	-0.02	0.15
Construction	1.1	8.23	7.13	2.16	1.06	9.97	8.87	4.71	3.61	3.15	2.05	-1.37	2.47
Trade etc.	2.45	0.12	2.33	-0.21	2.66	0.39	2.06	1.8	0.65	3.54	1.09	0.54	1.91
Financial activities etc.	2.21	2.51	0.3	1.54	0.67	2.91	0.7	2.21	0	4.4	2.19	0.18	2.03
Education etc.	0.2	0.2	0	-0.15	0.35	0.2	0	-0.44	0.64	-0.49	0.69	-0.73	0.93
Health etc.	-1.16	0.19	1.35	0.25	1.41	0.34	1.5	0.75	1.91	0.55	1.71	0.3	1.46
Personal services etc.	0.65	0.07	0.58	0.13	0.52	0.18	0.47	-0.19	0.84	0.54	0.11	-0.81	1.46
Public administration etc.	0.04	0.09	0.05	-0.58	0.62	0.05	0.01	-0.48	0.52	-0.36	0.4	8.11	8.07

Table S8. Change in percent points per 10 years over the period 2001-2013. Point estimates from linear regression and 95% confidence intervals obtained by boot-strapping. Statistically significant differences between each exposure and the population are marked in bold.

Variable	Population	RCS	Diesel exhaust	Welding	Wood dust	Chlorinated solvents	Lead
**Age**							
18-24	0.66	**3.21 (1.87 - 4.56)**	0.73 (0.11 - 1.36)	**2.07 (0.86 - 3.27)**	**2.6 (1.41 - 3.78)**	-0.11 (-1.22 - 0.99)	-0.08 (-0.94 - 0.78)
25-34	-1,93	-1.59 (-3.3 - 0.12)	**0.24 (-0.53 - 1.02)**	-2.59 (-4.29 - -0.9)	-1.73 (-3.13 - -0.32)	**-5.4 (-6.64 - -4.16)**	**2.02 (0.66 - 3.39)**
35-44	-0,65	**-4.04 (-5.47 - -2.62)**	**-5.18 (-5.81 - -4.56)**	**-4.04 (-5.45 - -2.64)**	**-3.11 (-4.06 - -2.15)**	**-2.28 (-3.79 - -0.77)**	**-2.2 (-3.11 - -1.29)**
45-54	0,59	**2.02 (0.83 - 3.2)**	**1.35 (0.75 - 1.95)**	**2.11 (1.43 - 2.78)**	1.64 (0.52 - 2.76)	**4.01 (3.11 - 4.91)**	**-1.02 (-2 - -0.03)**
>=55	1,32	0.41 (-0.83 - 1.64)	**2.86 (1.73 - 3.98)**	2.47 (1.2 - 3.74)	0.6 (-0.95 - 2.14)	**3.78 (3.02 - 4.55)**	1.27 (0.19 - 2.35)
**Sex**							
Females	-0,47	-0.66 (-1.2 - -0.12)	**1.28 (0.93 - 1.64)**	**0.25 (0.08 - 0.42)**	**-1.67 (-2 - -1.34)**	**-1.94 (-2.22 - -1.66)**	**4.44 (4.04 - 4.83)**
Males	0,47	0.66 (0.12 - 1.2)	**-1.28 (-1.64 - -0.93)**	**-0.25 (-0.42 - -0.08)**	**1.67 (1.34 - 2)**	**1.94 (1.66 - 2.22)**	**-4.44 (-4.83 - -4.04)**
**Country of birth**							
Sweden	-3,36	-2.69 (-3.41 - -1.97)	**-5.64 (-6.27 - -5)**	**-1.19 (-1.52 - -0.86)**	-3.48 (-4.21 - -2.75)	**-1.27 (-1.77 - -0.78)**	**-0.48 (-0.73 - -0.23)**
Other Nordic countries	-0,97	**-1.74 (-1.8 - -1.68)**	**-0.88 (-0.91 - -0.85)**	**-1.75 (-1.82 - -1.68)**	-0.93 (-0.97 - -0.9)	**-1.74 (-1.85 - -1.63)**	**-1.61 (-1.67 - -1.54)**
Other Europe	1,53	**2.85 (2.35 - 3.34)**	**2.16 (1.93 - 2.4)**	1.34 (1.12 - 1.57)	**2.8 (2.31 - 3.29)**	**1.26 (1.01 - 1.51)**	**0.81 (0.68 - 0.95)**
Outside Europe	2,79	**1.58 (1.32 - 1.85)**	**4.35 (3.93 - 4.78)**	**1.6 (1.42 - 1.77)**	**1.61 (1.38 - 1.85)**	**1.75 (1.56 - 1.94)**	**1.27 (1.14 - 1.4)**
**Company size**							
1-49 employees	3,07	**9.01 (7.4 - 10.62)**	**0.76 (-0.04 - 1.55)**	**4.65 (3.84 - 5.47)**	**10.22 (8.93 - 11.51)**	**7.95 (6.86 - 9.05)**	**0.27 (-0.88 - 1.43)**
50+ employees	-3,07	**-9.01 (-10.62 - -7.4)**	**-0.76 (-1.55 - 0.04)**	**-4.65 (-5.47 - -3.84)**	**-10.22 (-11.51 - -8.93)**	**-7.95 (-9.05 - -6.86)**	**-0.27 (-1.43 - 0.88)**
**Sector**							
Private	3,68	**1.19 (0.78 - 1.59)**	**1.19 (0.94 - 1.44)**	**1.49 (1.12 - 1.86)**	**1.89 (1.15 - 2.64)**	**-0.46 (-0.96 - 0.05)**	**-6.43 (-7.62 - -5.23)**
Public	-3,68	**-1.19 (-1.59 - -0.78)**	**-1.19 (-1.44 - -0.94)**	**-1.49 (-1.86 - -1.12)**	**-1.89 (-2.64 - -1.15)**	**0.46 (-0.05 - 0.96)**	**6.43 (5.23 - 7.62)**
**Industry branch**							
Not specified	-0,28	**1.08 (0.59 - 1.56)**	**-0.1 (-0.21 - 0.02)**	**0.17 (0 - 0.33)**	**0.98 (0.57 - 1.4)**	**0.15 (-0.04 - 0.33)**	**0.05 (-0.14 - 0.23)**
Agriculture etc.	-0,09	-0.15 (-0.34 - 0.03)	-0.18 (-0.38 - 0.01)	-0.04 (-0.13 - 0.06)	-0.15 (-0.36 - 0.05)	**0.23 (0.11 - 0.35)**	-0.05 (-0.11 - 0.01)
Manufacturing etc.	-5,26	**-12.49 (-14.07 - -10.9)**	**-2.99 (-4.41 - -1.58)**	**-8.63 (-9.53 - -7.72)**	**-14.95 (-16.58 - -13.31)**	**-11.74 (-12.73 - -10.74)**	-6.21 (-8.83 - -3.6)
Energy supply	0,13	0.15 (0.06 - 0.25)	0.13 (-0.03 - 0.3)	0.13 (0.04 - 0.22)	0.07 (-0.01 - 0.16)	0.04 (-0.1 - 0.17)	**-0.02 (-0.11 - 0.07)**
Construction	1,1	**8.23 (7.15 - 9.31)**	**2.16 (1.74 - 2.59)**	**4.71 (4.12 - 5.3)**	**9.97 (8.76 - 11.17)**	**3.15 (2.62 - 3.69)**	**-1.37 (-3.79 - 1.06)**
Trade etc.	2,45	**0.12 (-0.21 - 0.45)**	**-0.21 (-2.4 - 1.98)**	**1.8 (1.45 - 2.16)**	**0.39 (0.04 - 0.74)**	**3.54 (3.13 - 3.95)**	**0.54 (0.1 - 0.99)**
Financial activities etc.	2,21	2.51 (1.44 - 3.57)	**1.54 (1 - 2.08)**	2.21 (1.65 - 2.76)	2.91 (2.01 - 3.81)	**4.4 (3.87 - 4.94)**	**0.18 (-0.47 - 0.84)**
Education etc.	0,2	0.2 (0.11 - 0.29)	**-0.15 (-0.25 - -0.05)**	**-0.44 (-0.55 - -0.32)**	0.2 (-0.38 - 0.77)	**-0.49 (-1.09 - 0.11)**	**-0.73 (-0.91 - -0.54)**
Health etc.	-1,16	**0.19 (0.1 - 0.28)**	**0.25 (0.16 - 0.33)**	**0.75 (0.48 - 1.03)**	**0.34 (0.21 - 0.48)**	**0.55 (0.42 - 0.68)**	**0.3 (0.1 - 0.5)**
Personal services etc.	0,65	**0.07 (-0.18 - 0.32)**	**0.13 (0.03 - 0.23)**	**-0.19 (-0.62 - 0.24)**	**0.18 (-0.11 - 0.48)**	0.54 (0.37 - 0.7)	**-0.81 (-1.23 - -0.39)**
Public administration etc.	0,04	0.09 (0 - 0.18)	**-0.58 (-0.78 - -0.37)**	**-0.48 (-0.68 - -0.28)**	0.05 (-0.04 - 0.14)	**-0.36 (-0.49 - -0.24)**	**8.11 (6.69 - 9.54)**

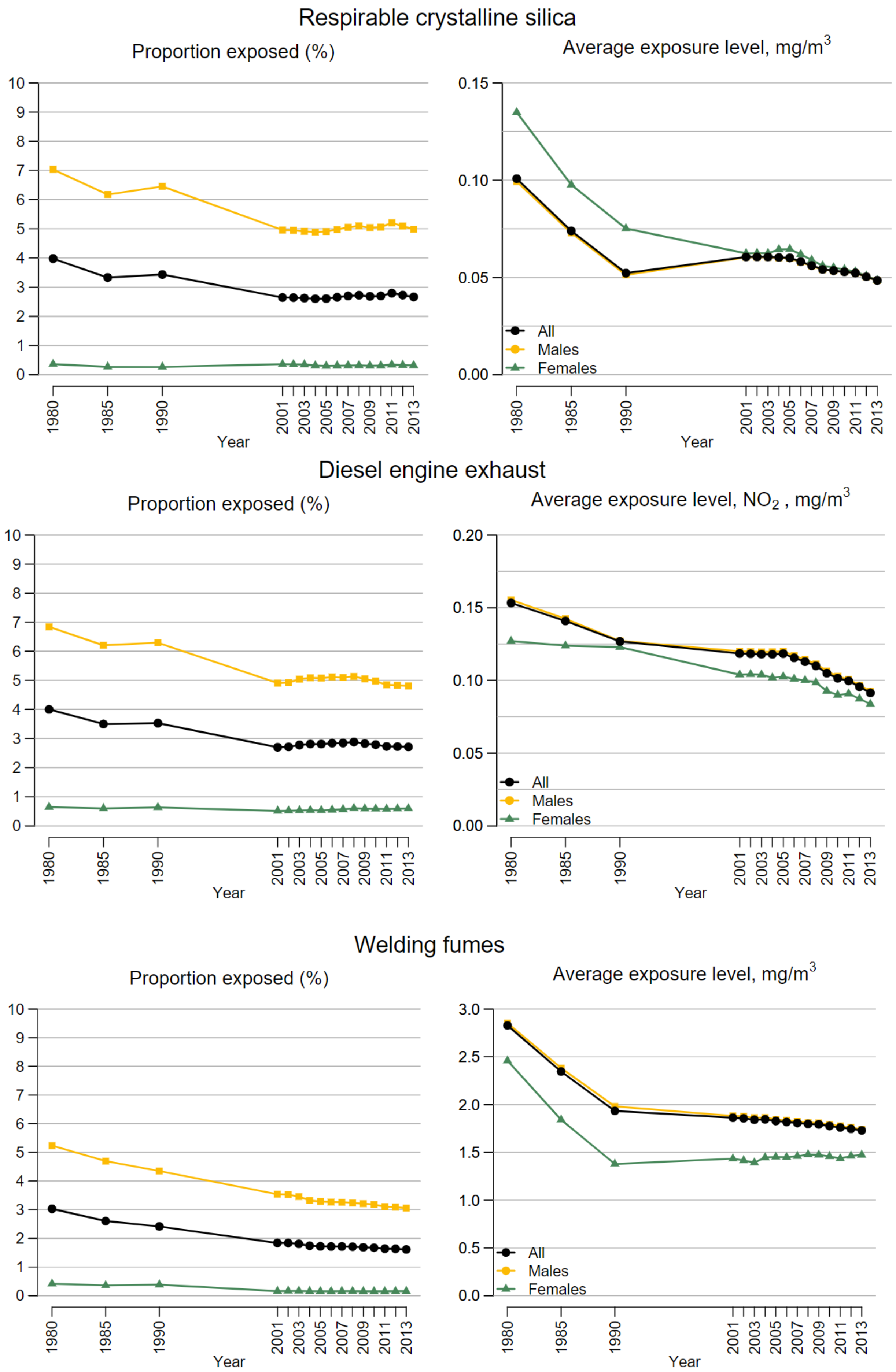

Fig S1. Time trends in proportions of the population being exposed to respirable crystalline silica, diesel engine exhaust and welding fumes, and the average exposure level in the exposed.

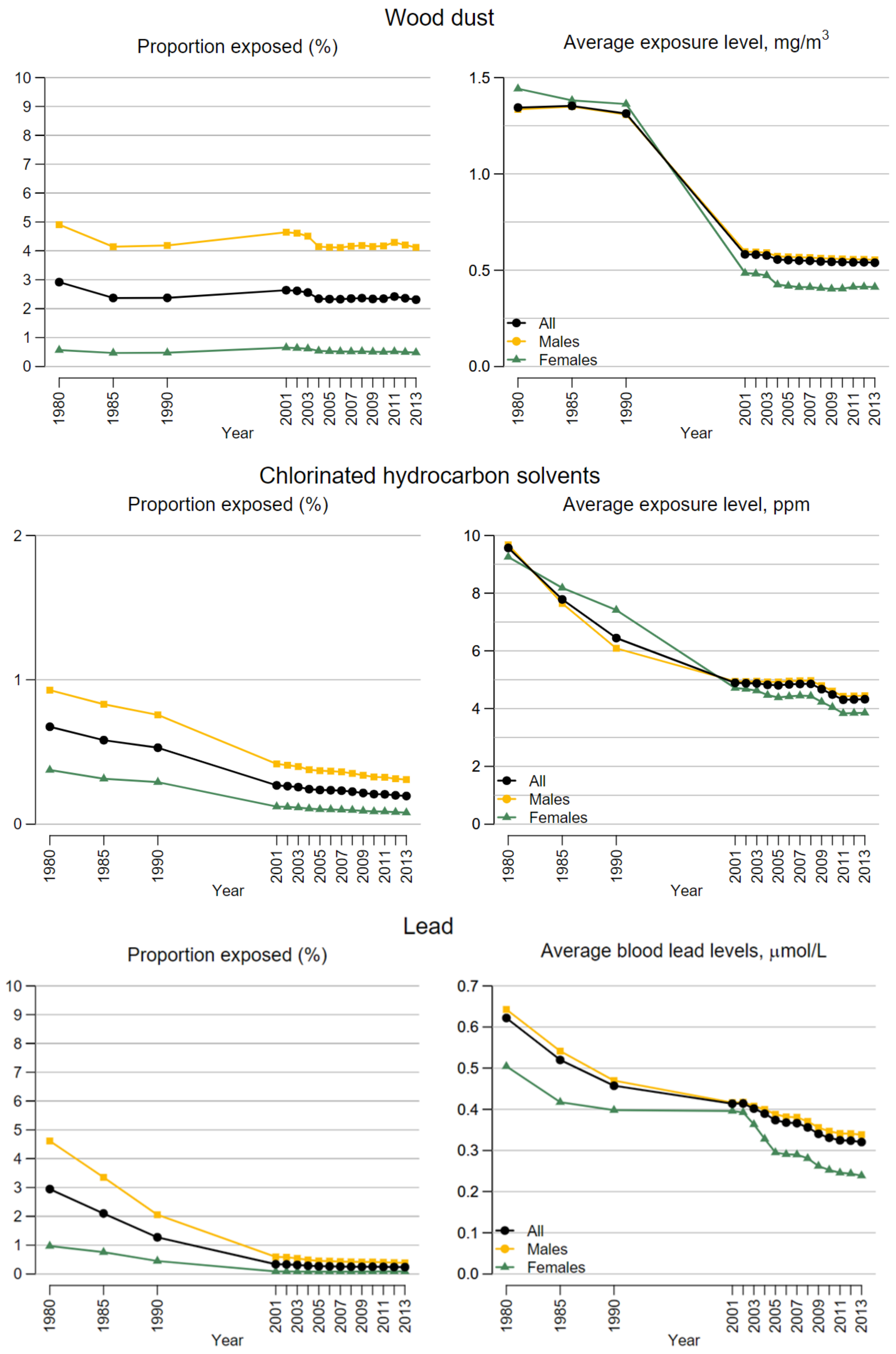

Fig S2. Time trends in proportion of the population being exposed to wood dust, chlorinated hydrocarbon solvents, and lead, and the average exposure level in the exposed.
